# Serving kinship families During COVID: Pivotal moments

**DOI:** 10.1093/geroni/igab046.3039

**Published:** 2021-12-17

**Authors:** Andrea Smith, Michelle Mongeluzzo, Tawyna Drente

**Affiliations:** 1 Western Michigan University, Ada, Michigan, United States; 2 Kids Central Inc, Wildwood, Florida, United States

## Abstract

Kinship caregivers, who are relatives or non-family members providing care to children when biological parents are unable to do so, comprise over 2.5 million adults in the United States. The vast majority are grandparent caregivers. The 7.8 million children in their care make up approximately 10.5 percent of all children in the United States under the age of 18 (Generations United, 2017: State of Grandfamilies). Navigating daily life is often challenging. Kinship caregivers routinely face difficulties in multiple aspects of their lives, including finances, physical health, mental health, education, employment, parenting, and family relationships. The COVID pandemic heightened existing challenges and stimulated new issues for many kinship providers and the children in their care. This poster will highlight actions taken by one Family Service agency, annually serving approximately 225 kinship families, to meet the unprecedented needs of family members and kinship program staff during COVID. A timeline of decision-related rationales, specific actions taken and results related to these actions will be presented. Data summarizing results for kinship families (n =32) related to COVID-impacted programmatic responses and changes, including level of involvement with group services, recidivism, perceived isolation, and efficacy related to their caregiving roles will be presented. Results summarizing the impact of the agency's COVID-related responses on kinship staff (n = 6) will also be presented, including data on staff members' level of stress, perceived support, perceptions of programmatic effectiveness, and prioritized importance of changes will also be shared.

